# Extragastrointestinal Stromal Tumor: A Differential Diagnosis of Compressive Upper Abdominal Tumor

**DOI:** 10.1155/2018/1052960

**Published:** 2018-05-09

**Authors:** Clara Kimie Miyahira, Miguel Bonfitto, Jéssyca Fernanda de Lima Farto, Annelise de Figueiredo Calili, Nathalia Rabello da Silva Sousa, Ana Paula de Figueiredo Calili

**Affiliations:** ^1^São José do Rio Preto Medical School, São José do Rio Preto, SP, Brazil; ^2^Hospital de Base de São José do Rio Preto, São José do Rio Preto, SP, Brazil; ^3^Marília Medical School, Marília, SP, Brazil

## Abstract

**Introduction:**

Extragastrointestinal stromal tumors (EGIST) are rare mesenchymal tumor lesions located outside the gastrointestinal tract. A rare compressing tumor with difficult diagnosis is reported.

**Presentation of the Case:**

A male patient, 63 years old, was admitted in the emergency room complaining of stretching and continuous abdominal pain for one day. He took Hyoscine, with partial improvement of symptoms, but got worse due to hyporexia, and the abdominal pain persisted. The patient also reported early satiety and ten-pound weight loss over the last month.

**Discussion:**

EGIST could be assessed by CT-guided biopsy, leading to diagnosis and proper treatment with surgical resection or Imatinib.

**Conclusion:**

This case report highlights the importance of considering EGIST an important differential diagnosis of compressing upper abdominal tumors.

## 1. Introduction

Gastrointestinal stromal tumors (GIST) are rare lesions in the mesenchymal neoplasm, accounting for less than 1% of the primary neoplasias of the digestive tract. They may affect any segment of the gastrointestinal tract but can occur in other locations in only 10% of the cases, and, in these situations, they are called EGIST [1]. The diagnosis is hard and may be made through CT-guided puncture and immunohistochemical analysis of the biopsy.

There are three histological types: spindle (70%), epithelial (20%), and mixed-cell. In 95%, there is somatic mutation of CD117 (c-kit), and its discovery in the immunohistochemical characteristic defines the GIST [2]. Staging could be done with abdominal and pelvis tomography, MRI, or PET-CT [3].

A case of massive EGIST is reported to show a rare differential diagnosis of an upper abdominal tumor, emphasizing the proper treatment due to correct diagnosis.

## 2. Presentation of the Case

A male patient, 63 years old, was admitted in the emergency room complaining of stretching and continuous abdominal pain for one day. He took Hyoscine, with partial improvement of symptoms, but got worse due to hyporexia, and the abdominal pain persisted for a few hours after medication. The patient also reported early satiety and ten-pound weight loss over the last month.

He is a smoker for nearly fifty years. He has no other comorbidities, previous surgeries, nor family history. In the physical examination, the patient showed flat abdomen, pain on superficial palpation of the epigastrium, no rebound tenderness, and palpable mass approximately 12 cm wide in the left abdominal quadrant.

**Figure 1:**
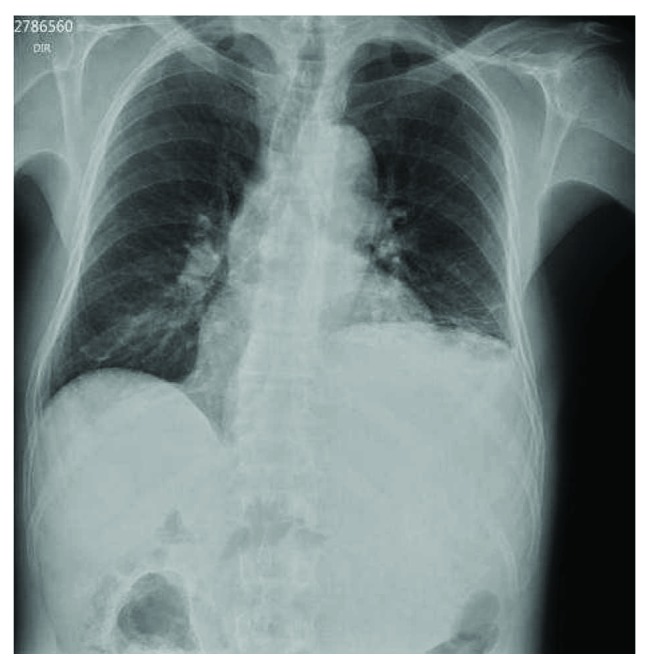
Acute abdominal X-ray showing the chest with elevated left hemidiaphragm.

**Figure 2:**
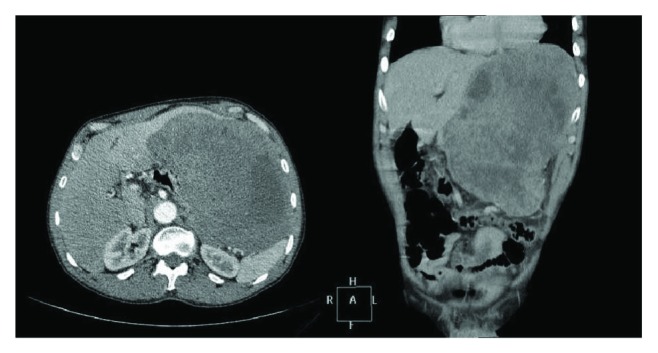
Contrast-enhanced computed tomography showing wide hypodense mass in the left hemiabdomen.

The laboratory assessment was normal. An acute abdominal radiography (Figure 1) was taken, showing elevated left hemidiaphragm. The abdominal tomography (Figure 2) showed a wide hypodense mass with necrosis and heterogeneous absorption. The mass was posterior to the stomach and adjacent to the spleen and left kidney, without a cleavage plane between the left lobe of the liver and the pancreatic body, also compressing adjacent organs, invading the posterior wall of the stomach. The patient also underwent upper digestive endoscopy, showing bulging and gastric mucosal edema.

**Figure 3:**
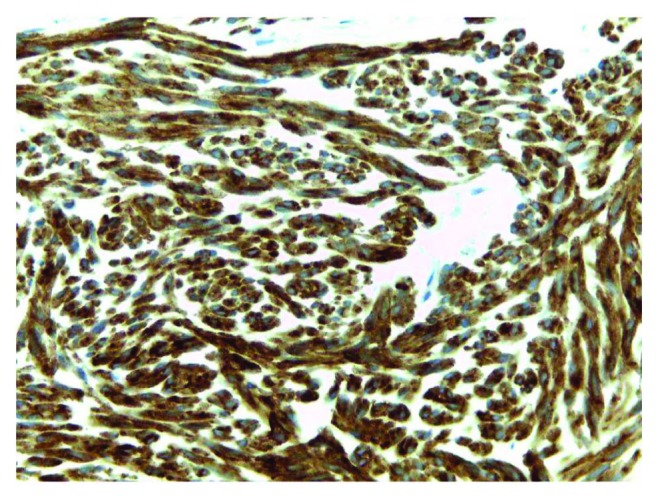
Immunohistochemical analysis positive for CD117 (C-kit) (original magnification 40x).

A CT-guided biopsy was taken, resulting in immunohistochemical analysis positive for C-Kit (Figure 3), CD34, and Ki67. These findings led to the correct diagnosis of extragastrointestinal stromal tumor (EGIST). The abdominal tomography was performed in the emergency room, also suggesting this type of tumor. The EGIST was a T4N0M0. As the tumor was greater than 2 cm and nonresectable, surgery was not suggested. The neoadjuvant therapy started with Imatinib, with weekly clinical follow-up.

## 3. Discussion

The EGIST is a rare diagnosis regarding stromal tumors and can affect other locations in addition to the gastrointestinal tract, such as the omentum, pancreas, rectum, and small intestine. It is an important differential diagnosis of masses in the upper abdomen: leiomyoma, leiomyosarcoma, lipoma, schwannoma, carcinoids, and fibroids. CT-guided puncture or ultrasound may provide biopsy material. The immunohistochemical analysis with CD117 (C-Kit) confirms the diagnosis [4–6]. A recent study showed positive immunohistochemistry in 93.3% for CD117, 70% for CD34, and 10% for S1007. The biopsy result in this case report was positive for CD117, CD34, and Ki67.

Once the diagnosis is established, it is necessary to stage the tumor for better management [7]. The study patient was staged as T4N0M0 and nonresectable. The guidelines suggest that the first-line systemic treatment for advanced GIST cases is neoadjuvant therapy with Imatinib [3, 8, 9]. This treatment may result in 83–89% of patients responding or having the progression of the disease stabilized [8].

## 4. Conclusion

The EGIST should be noted as an important diagnosis of tumor masses, especially when symptomatic, such as masses in the upper abdomen. The correct diagnosis is very relevant, to the extent that it outlines the choice of clinical or surgical treatment.

## Abbreviations

EGIST: Extragastrointestinal stromal tumors
GIST: Gastrointestinal stromal tumors.
